# Changes in dream features across the first and second waves of the Covid‐19 pandemic

**DOI:** 10.1111/jsr.13425

**Published:** 2021-06-22

**Authors:** Francesca Conte, Marissa Lynn Rescott, Oreste De Rosa, Nicola Cellini, Alessia Coppola, Mariangela Cerasuolo, Serena Malloggi, Fiorenza Giganti, Gianluca Ficca

**Affiliations:** ^1^ Department of Psychology University of Campania L. Vanvitelli Caserta Italy; ^2^ Department of General Psychology University of Padova Padova Italy; ^3^ Department of Biomedical Sciences University of Padova Padova Italy; ^4^ Padova Neuroscience Center University of Padova Padova Italy; ^5^ Human Inspired Technology Center University of Padova Padova Italy; ^6^ Department NEUROFARBA University of Florence Florence Italy

**Keywords:** Covid‐19 pandemic, dream affect, dream length, dream recall frequency, dream vividness

## Abstract

Research during the Covid‐19 pandemic has highlighted its significant impact on dreaming. Here we address changes in dream features both during the first wave, when the Italian government imposed a total lockdown, and the second wave (autumn 2020), when a partial lockdown was effected. In April 2020 (total lockdown), 1,622 participants (M_age_ = 34.1 ± 13.6 years; 1171F) completed an online survey including the Pittsburgh Sleep Quality Index and a set of questions on dream features and their possible changes relative to the month preceding the lockdown (pre‐total lockdown). In November 2020 (partial lockdown), 214 participants (M_age_ = 36.78 ± 14.2 years; 159F) from the previous sample completed the same survey. Approximately half of the subjects reported increased or decreased dream frequency (30.5% and 21.8%), length (27.1% and 15.8%) and vividness (31.5% and 17.1%) during total lockdown as well as during partial lockdown (frequency: 30.3% and 13.5%; length: 23.3% and 12.6%; vividness: 31.6% and 24.1%). Dream affect became significantly more negative in total lockdown relative to pre‐total lockdown and in partial lockdown relative to pre‐partial lockdown (both *p* < .001). Both in total lockdown and partial lockdown, increased negative dream emotionality significantly predicted changes in dream frequency, length and vividness, and was significantly predicted, in turn, by worsened sleep quality. Our data confirm that dream features are significantly affected by major life changes such as those imposed by a pandemic. The fact that between lockdowns negative dream affect returned almost to baseline level suggests that dream emotionality is closely related to lifestyle and wake‐time emotional changes. Also, our findings point to a modulating role of sleep quality on dream emotionality.

## 1 INTRODUCTION

In March 2020, Italy became the first western country to face a public health emergency related to the COVID‐19 pandemic. By 9 March 2020, more than 8,000 people had contracted the virus, and the Italian government responded by expanding a lockdown of the northern provinces to the nationwide lockdown that would go on to last more than 2 months (Istituto Superiore di Sanità, 2020).

The Ministerial Decrees signed by Italian President Giuseppe Conte forbade gathering of people in public places, restricted movement within their towns, and closed all commercial/retail activities, while allowing grocery and basic necessities stores, along with pharmacies, to remain open (Governo Italiano, Presidenza del Consiglio dei Ministri, 2020). People living in Italy were advised to avoid leaving the house except for health reasons or to purchase necessary goods, and were required to carry self‐declaration documentation to justify their reason for leaving home; this decree was enforced by police checks, and dishonesty in documentation was criminalized and punished with a fine (Governo Italiano, Presidenza del Consiglio dei Ministri, 2020).

The public emergency itself as well as the substantial changes in daily routines had a significant psychological impact on the Italian population (Cellini, Canale, Mioni, & Costa, 2020; Moccia et al., 2020; Rossi et al., 2020). Generally, the public reported decreased levels of psychological wellbeing, especially people with pre‐existing psychiatric conditions, COVID‐19 patients, and healthcare workers (Vindegaard & Benros, 2020). Surveys administered early in the lockdowns suggested that an increased percentage of people in Italy were reporting high and very high levels of distress compared with European norms (Moccia et al., 2020). Another study revealed similar findings, specifically relatively higher rates of: post‐traumatic stress symptoms, depression symptoms, anxiety, perceived stress and adjustment disorder symptoms (Rossi et al., 2020). Risk factors for higher levels of psychological distress included female gender, anxious or depressive temperaments, and discontinued working activity (Moccia et al., 2020; Rossi et al., 2020).

The pandemic lockdowns also resulted in adverse effects on sleep habits and sleep quality. Over 40% of an Italian sample reported sleep disturbances during the last 2 weeks of the lockdown period and, within that group, 17.4% reported moderate to severe insomnia (Gualano, Lo Moro, Voglino, Bert, & Siliquini, 2020). We described previously that during home confinement, Italians reported delayed bed/wake times and increased time spent in bed as well as an impairment in sleep quality (Cellini et al., 2020, 2021). Italian women and youth were found to be at greater risk for distress and sleep disorders as a result of COVID‐19 (Casagrande, Favieri, Tambelli, & Forte, 2020; Cellini et al., 2021). Similar findings on sleep changes have been reported in Argentinian, Australian, Belgian, Chinese, French and Greek populations, suggesting global impact (Cellini et al., 2021; Huang & Zhao, 2020; Kokou‐Kpolou, Megalakaki, Laimou, & Kousouri, 2020; Leone, Sigman, & Golombek, 2020; Stanton et al., 2020; Voitsidis et al., 2020; Xiao, Zhang, Kong, Li, & Yang, 2020).

To date, research groups in Brazil, Canada, China, Finland, Italy and USA have also investigated how dreams were impacted by COVID‐19. These groups largely found that participants reported specific dream content related to the pandemic, including disregard for social distancing, loved ones becoming sick, biological processes, death, and changes in location (Barrett, 2020; MacKay & DeCicco, 2020; Parrello, Sommantico, Lacatena, & Iorio, 2020; Pesonen et al., 2020). Pandemic‐specific dream content was associated with female gender, increased dream recall, increased perceived stress, and was more frequent in individuals who were most “affected” by the pandemic (Barrett, 2020; Parrello et al., 2020; Schredl & Bulkeley, 2020). In addition to changes in dream content, dream researchers also found higher levels of dream anxiety, increasingly negative dream emotionality, and more frequent nightmares (Barrett, 2020; Gorgoni et al., 2021; Iorio, Sommantico, & Parrello, 2020; Scarpelli et al., 2021; Schredl & Bulkeley, 2020; Xu, Xu, Wang & Wang 2020). Similar to the sleep quality and dream content findings, females and those who were most strongly affected by the virus (physically and/or psychologically) were more likely to report the emotionality changes in dreaming (Barrett, 2020; Gorgoni et al., 2021; Iorio et al., 2020; Scarpelli et al., 2021; Schredl & Bulkeley, 2020; Xu et al., 2020). However, men also reported elevated rates of negative emotions, anxiety, and death in their dreams, though at a lower level of significance (Barrett, 2020). Unsurprisingly, both depression and anxiety scores were correlated with dream anxiety (Xu et al., 2020) and nightmare frequency (Scarpelli et al., 2021), further underlining the impact of the pandemic on mental health in both wake and sleep.

The research reviewed above was performed during the first wave of the COVID‐19 pandemic that abated in May. Then, from June to September, lockdown restrictions in Italy were relaxed, travel within Europe had opened, and people had begun moving around again (Governo Italiano, Presidenza del Consiglio dei Ministri, 2020). However, starting in September, a second and larger wave of infections began to spread through the country and, by November, the number of daily new cases (~34,000 positive cases per day) had increased by over five times the number reported during the height of the pandemic in March (~6,500 positive cases per day; Governo Italiano, Presidenza del Consiglio dei Ministri, 2020). This time, the government responded with a graded and partial lockdown (PL) based on the level of cases within each region of the country (Governo Italiano, Presidenza del Consiglio dei Ministri, 2020).

There exist multiple possibilities for how the Italian population may have responded psychologically to this timeline due to the magnitude of life changes that have occurred as a result of lockdowns. One possibility is that the population emotionally habituated to the pandemic conditions following the first wave, resulting in improved wellbeing that lasted through the summer and fall, and increased resilience against the psychological effects of a second wave of infection. Another possibility is that following the first wave of the pandemic, during the period of eased restrictions, the people of Italy experienced improved wellbeing, then experienced a more severe decline in wellbeing once the second, and more severe, wave overtook the country. Indeed, in late October and November, people protested against another lockdown in a number of Italian cities, suggesting that the economic effects of lockdowns may have been more damaging than the fear of contagion (Covid: Protests take place across Italy over anti‐virus measures ‐ BBC News).

Given that many of the Italian population's psychological health, sleep and dream patterns had undergone significant changes during the first wave of the COVID‐19 pandemic when the daily cases were relatively low, we aimed to investigate the similarities and differences in those same outcomes during the second wave of the pandemic. To our knowledge, this is the first study to investigate the stability of dream data over time during a pandemic. Importantly, our study investigates dream features before and during both the first and second COVID‐19 waves.

## 2 MATERIALS AND METHODS

### 2.1 Participants and procedure

During the first wave of the pandemic, a sample of 1,622 participants (age range: 18–79 years, mean age = 34.1 ± 13.6 years; 1171 F) residing in Italy completed an anonymous online survey, the “COVID‐19 Questionnaire on Sleep Habits”, from 1 April to 20 April 2020, advertised across the whole nation via social media and University websites. The only inclusion criterion was age > 18 years.

Before completing the questionnaire, participants were asked to read the aims of the study and to explicitly agree to participate in the survey by filling in the consent form. The survey took approximately 25 min to be completed. There was no money or credit compensation for participating in the study.

At the end of the survey, participants were asked to leave a contact (e‐mail address) if they were willing to be re‐contacted for a further assessment of their sleep habits. Out of the 1,622 participants, 443 provided consent to take part in the study follow‐up. These participants were contacted again on 10 November with the request to complete a slightly modified and abbreviated version of the first survey. A reminder was sent after 1 week and after 2 weeks; data collection was ended on 1 December. The final sample of the follow‐up survey consists of 214 participants (age range: 18–73 years, mean age = 36.1 ± 14.2 years; 159 F).

The Ethical Committee of the Department of Psychology, University of Campania “Vanvitelli”, approved the research protocol. All methods were carried out in accordance with relevant guidelines and regulations.

Data reported here are part of a wider research project designed to assess several aspects of sleep during the lockdown; other data with different research purposes are presented elsewhere (Cellini et al., 2021).

### 2.2 The instrument

The survey administered during the total lockdown (TL, spring 2020) was made up of several sections, containing questions on: current working conditions and daily habits, current problems and worries related to the ongoing COVID‐19 epidemic, health status, depressive and anxiety symptoms, sleep and dreams. The section on sleep included the Italian version of the Pittsburgh Sleep Quality Index (PSQI; Curcio et al., 2013), and a few additional questions on night awakenings and sleep‐related habits. Past sleep habits and characteristics were also investigated through retrospective questions referring to the month preceding the lockdown (BEFORE).

Dreaming activity, which is the main object of this study, was specifically investigated using six questions: three regarded the possible modification of dream frequency, length and vividness after the lockdown (e.g. “Referring to your current situation [NOW], how often do you recall your dreams?” “More often than BEFORE”, “Less often than BEFORE”, “The same”); one regarded prevalent emotional valence of current (NOW) and past (BEFORE) dreams (e.g. “What is the prevalent emotional tone of your dreams?” “Very positive”, “Moderately positive”, “Neutral”, “Moderately negative”, “Very negative”); two referred to the possible occurrence of dream content related to the epidemic or the general state of emergency (i.e. “Referring to your current situation [NOW], do you happen to dream of events in some way related to the COVID‐19 epidemic or to the general state of emergency?” “Yes”, “No”; “If so, in these dreams do you happen to face problematic situations that you are currently coping with in your daily life?” “Yes”, “No”).

As for psychological variables, they were assessed through single items regarding mood (“In the current situation, how is your prevalent mood?” “Very Positive”, “Moderately Positive”, “Neutral”, “Moderately Negative”, “Very Negative”), stress (“In the current situation, how stressed do you generally feel?” “Not at all stressed”, “Moderately stressed”, “Extremely stressed”), general fear (“In the current situation, how afraid do you generally feel?” “Not at all afraid”, “Moderately afraid”, “Extremely afraid”), fear of Covid‐19 contagion (“In the current situation, how afraid do you feel of being personally infected or that any of your dear ones could be infected?” “Not at all afraid”, “Moderately afraid”, “Extremely afraid”). Depression and anxiety symptoms were also investigated through single items (“In the current situation, how often do you happen to feel depressed for most of the day?” “Often”, “Sometimes”, “Never”; “In the current situation, how often do you happen to feel very anxious or to panic?” “Often”, “Sometimes”, “Never”).

The same survey was administered as a follow‐up during the PL (autumn 2020).

### 2.3 Data analysis

Because of the different sample sizes between the two time‐points of data collection, separate analyses were conducted on dream measures collected during TL (*n* = 1622) and PL (*n* = 214).

The acronyms “pre‐TL” and “pre‐PL” refer to the month preceding the TL and PL, respectively.

Non‐parametric statistics was used due to non‐normal distribution of variables. Differences in dream emotional valence between the lockdowns and the preceding months were assessed through the Wilcoxon rank test. Instead, Mann–Whitney test was employed to evaluate differences in dream valence change (Δ valence, i.e. emotional valence score NOW minus emotional valence score BEFORE) between those subjects who reported Covid‐19‐related dreams and those who did not.

Multinomial logistic regressions were performed to investigate predictors of changes (increased and decreased) in dream frequency, length and vividness, with “equal” (no change during the lockdown) as reference. Predictors included in the models were: demographics (age and gender, with male as reference), psychological variables (mood, with “neutral” as reference; stress, general fear, fear of contagion, with “not at all stressed/afraid” as reference), change in sleep quality (ΔPSQI, i.e. PSQI score NOW minus PSQI score BEFORE), change in sleep midpoint (Δsleep midpoint, i.e. sleep midpoint NOW minus sleep midpoint BEFORE) and change in dream emotional valence (Δvalence).

Predictors of changes in dream emotional valence (Δvalence) were assessed through a multiple linear regression, considering demographics (age and gender, with male as reference), psychological variables (mood, with “neutral” as reference; stress, general fear, fear of contagion, with “not at all stressed/afraid” as reference), changes in sleep quality and sleep midpoint (ΔPSQI and Δsleep midpoint) as predictors.

Finally, a binary logistic regression was used to investigate predictors of the presence of Covid‐19‐related dreams. Predictors included in the models were: demographics (age and gender, with male as reference), psychological variables (mood, with “neutral” as reference; stress, general fear, fear of contagion, with “not at all stressed/afraid” as reference), change in sleep quality and sleep midpoint (ΔPSQI and Δsleep midpoint), change in dream emotional valence (Δvalence), change in dream frequency, length and vividness (with “equal” as reference).

For the logistic regressions, we reported the *χ* ^2^, the *p*‐value and Nagelkerke's *R* ^2^ for the overall model, the unstandardized (*b*) coefficient and the odds ratio (OR) with 95% confidence interval (CI) of the significant predictors. For the linear regression, we reported the *F*‐ and *p*‐values and the adjusted *R* ^2^ for the overall model, and the *t*‐value and the standardized (*β*) coefficient for each predictor.

All analyses were conducted using JAMOVI 1.8.1, and statistical significance was set at *p* ≤ .05.

## 3 RESULTS

### 3.1 Sleep quality and psychological measures

Average PSQI scores were 5.20 ± 2.68 in pre‐TL, 6.56 ± 3.63 in TL, 5.28 ± 2.93 in pre‐PL and 6.02 ± 3.03 in PL, indicating worsened sleep quality in both lockdowns compared with the preceding periods.

As for psychological measures, Table 1 displays the frequency distribution in our sample of mood, general fear, fear of contagion and stress levels during TL and PL. In order to have a single overall index for each of these variables, we also calculated, at the two time‐points, their average scores. For mood, average scores were computed on a 0–4 scale, considering 0 as very positive mood, 1 as moderately positive mood, 2 as neutral mood, 3 as moderately negative mood, and 4 as very negative mood. For the other three variables, a 0–2 scale was used, with 0 as absence of fear/stress, 1 as presence of moderate fear/stress, 2 as presence of extreme fear/stress. For all four variables, average scores were slightly higher in PL relative to TL (mood: 2.17 ± 0.99 versus 1.48 ± 1.32; general fear: 0.90 ± 0.49 versus 0.82 ± 0.55; fear of contagion: 1.29 ± 0.53 versus 1.17 ± 0.78; stress: 1.02 ± 0.48 versus 0.95 ± 0.57, respectively), indicating worsened mood and higher levels of fear and stress during PL. This is consistent with the strikingly higher percentage, in PL, of participants who reported to know someone who was positive to Covid‐19 (37.48% in TL versus 91.09% in PL).

**TABLE 1:** Frequency distribution of mood, fear and stress levels during TL and PL

	TL	PL
Mood		
Very negative	4.7%	7.9%
Moderately negative	29.8%	33.6%
Neutral	29.8%	1.9%
Moderately positive	31.2%	29%
Very positive	4.4%	27.6%
General fear		
Extreme	11.1%	7.9%
Moderate	68.2%	74.2%
No fear	20.7%	17.7%
Fear of contagion		
Extreme	36.1%	33.1%
Moderate	56.3%	63%
No fear	7.6%	3.4%
Stress		
Extreme	14.2%	13%
Moderate	67.3%	76.1%
No stress	18.5%	10.7%

PL, partial lockdown; TL, total lockdown.

Psychological measures were also analysed longitudinally on the 214 participants who responded to the follow‐up survey, using the Wilcoxon signed‐rank test to assess differences in these variables between TL and PL. Participants showed similar mood (2.09 ± 1.02 in TL versus 2.17 ± 0.99 in PL; *W* = 5831, *p* = .508) and stress levels (0.96 ± 0.60 in TL versus 1.02 ± 0.48 in PL; *W* = 966, *p* = .100) during the two lockdowns. Instead, general fear for the pandemic situation (0.82 ± 0.56 in TL versus 0.90 ± 0.49 in PL; *W* = 722, *p* = .045) and fear of Covid‐19 contagion (1.17 ± 0.58 in TL versus 1.29 ± 0.53 in PL; *W* = 876, *p* = .003) turned out to be significantly higher in PL than TL.

### 3.2 Changes in dream recall frequency, dream length and dream vividness in TL and PL

**FIGURE 1:**
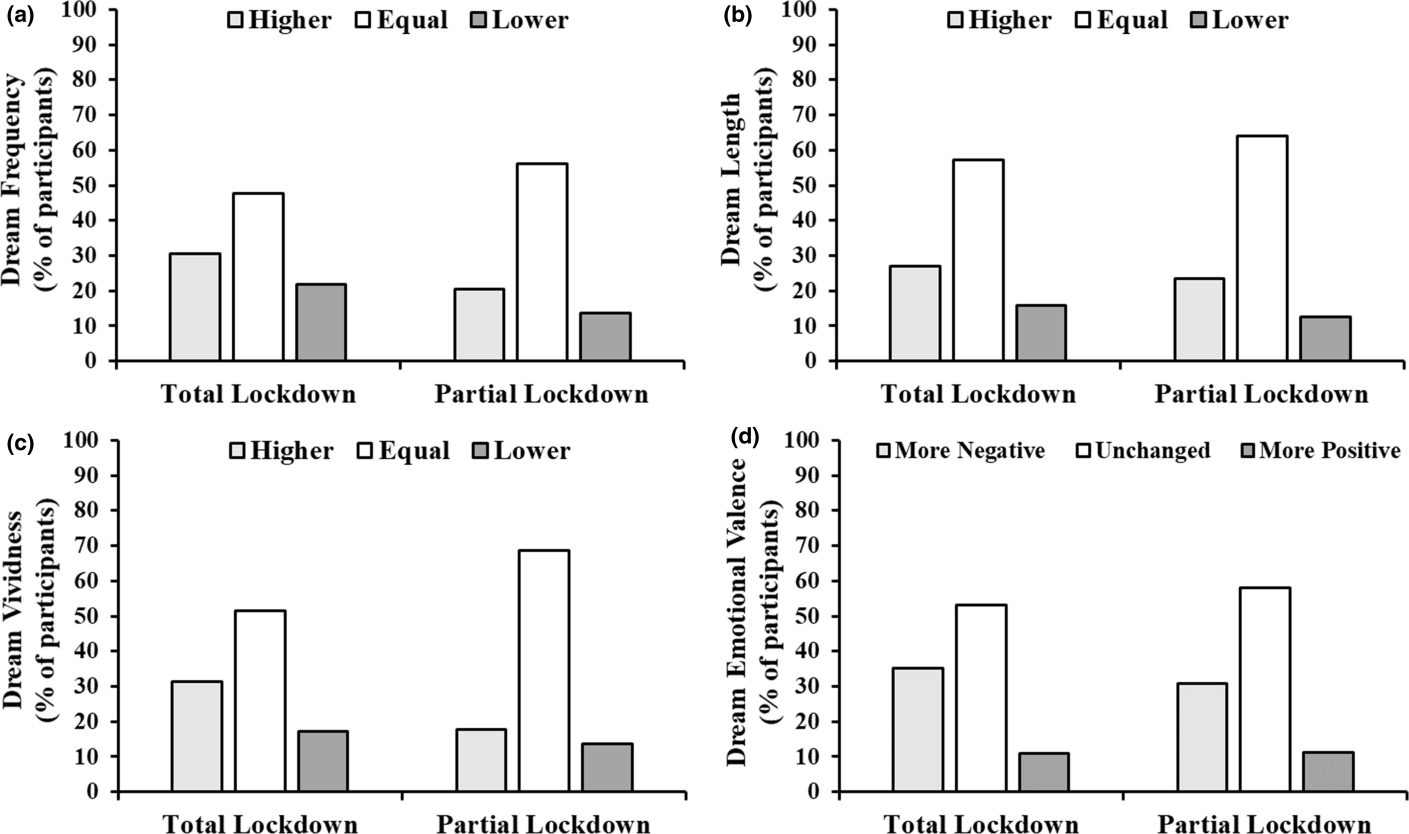
Proportion of subjects reporting higher/lower/equal dream frequency (a), length (b), vividness (c), and more negative/unchanged/more positive dream emotional valence (d) during total lockdown (TL) and partial lockdown (PL) relative to the previous months (pre‐TL and pre‐PL)

During TL, approximately half of the subjects reported increased or decreased dream frequency (increased: *n* = 494, 30.5%; equal: *n* = 775, 47.8%; decreased: *n* = 353, 21.8%), length (increased: *n* = 439, 27.1%; equal: *n* = 927, 57.2%; decreased: *n* = 256, 15.8%) and vividness (increased: *n* = 511, 31.5%; equal: *n* = 836, 51.5%; decreased: *n* = 275, 17.1%) compared with before the emergency (Figure 1a–c). A similar profile emerged during PL, with about 40% of the sample reporting increased or decreased dream frequency (increased: *n* = 65, 30.3%; equal: *n* = 120, 56.1%; decreased: *n* = 29, 13.5%), and about 30% reporting changes in dream length (increased: *n* = 50, 23.3%; equal: *n* = 137, 64.1%; decreased: *n* = 27, 12.6%) and vividness (increased: *n* = 38, 17.8%; equal: *n* = 147, 68.7%; decreased: *n* = 29, 13.6%; Figure 1a–c).

### 3.3 Changes in dream emotional valence in TL and PL

**FIGURE 2:**
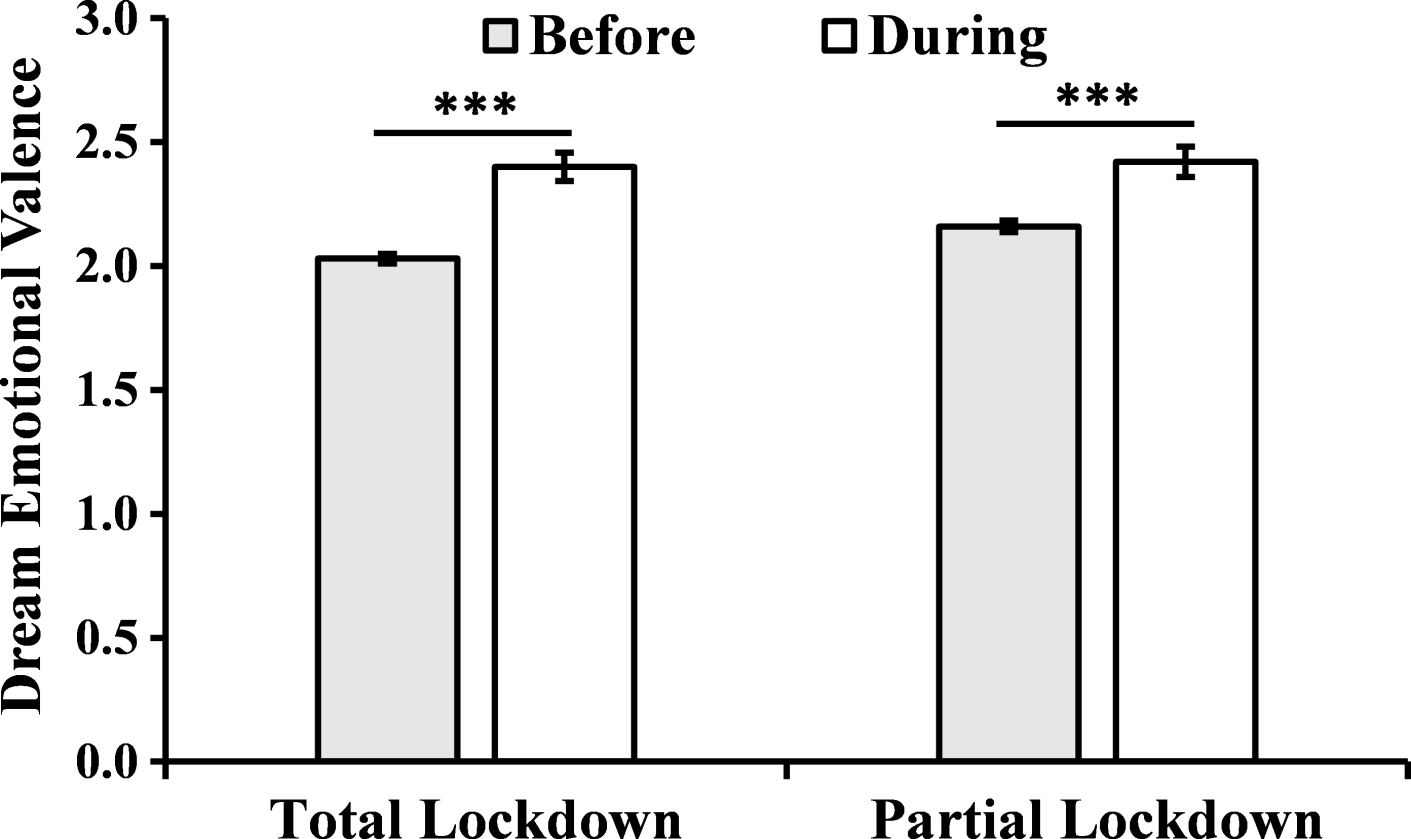
Changes in dream emotional valence (range 0–4) from pre‐total lockdown (TL) to TL, and from pre‐partial lockdown (PL) to PL. Higher scores indicate more negative dream emotionality

As displayed in Figure 2, dream emotional valence became significantly more negative in TL (2.31 ± 0.91) relative to pre‐TL (1.96 ± 0.77; *W* = 60663, *p* < .001), as well as in PL (2.42 ± 0.89) relative to pre‐PL (2.16 ± 0.83; *W* = 1137, *p* < .001). The proportion of subjects reporting changes in dream emotional valence during TL and PL compared with the months preceding them is displayed in Figure 1(d).

### 3.4 Presence of COVID‐19‐related dreams in TL and PL

During TL (*n* = 1,622), 25% of subjects reported having Covid‐19‐related dreams and, among these, 70% reported to face, in these dreams, problematic situations similar to those of their daily life. Very similar proportions were found during PL, with COVID‐19‐related dreams reported by 25.7% of the subjects and, among these, 80% reporting to face, in these dreams, problematic situations similar to those of their daily life.

Both during TL and PL, participants who reported having dreams related to Covid‐19 also showed a more pronounced shift to negative dream emotional valence (Δvalence score) compared with those who did not report Covid‐19‐related dreams (TL: *U* = 3,413; *p* < .001; PL: *U* = 3,338; *p* = .003).

### 3.5 Longitudinal assessment of dream features across the four time‐points

Longitudinal analyses were also conducted on the 214 participants who responded to the follow‐up survey.

Differences in dream emotional valence across the four time‐points (pre‐TL, TL, pre‐PL and PL) were assessed using the Friedman test and the Durbin–Conover test as post hoc analysis. Significant differences across the four periods emerged (*Χ* ^2^_3_ = 48.74, *p* < .001), with an increase of negative emotionality in TL (2.41 ± 0.80, *p* < .001) relative to pre‐TL (2.04 ± 0.86), and a reduction in pre‐PL (2.16 ± 0.84) compared with TL (*p* < .001), followed by another increase in PL (2.42 ± 0.89, *p* < .001).

To assess the differences between TL and PL in the frequency distribution of participants who experienced an increase, decrease or no change in dream features (frequency, length and vividness), we used McNemar's test. Significant differences emerged between lockdowns for all three variables. As for dream frequency (*Χ* ^2^_3_ = 13.73, *p* = .003), in PL compared with TL, we found fewer participants reporting an increase (*n* = 85, 39.7% in TL; *n* = 65, 30.3% in PL) or a decrease (*n* = 43, 20.0% in TL; *n* = 29, 13.5% in PL), and more participants reporting no change in this variable (*n* = 86, 40.19% in TL;/, *n* = 120, 56.1% in PL). Regarding dream length (*Χ* ^2^_3_ = 11.28, *p* = .010), the proportion of participants reporting a decrease in this variable remained stable (*n* = 27, 12.6% in both TL and PL), whereas in PL relative to TL fewer participants reported an increase (*n* = 79, 36.9% in TL; *n* = 50, 23.3% in PL), and more numerous participants reported no change (*n* = 108, 50.5% in TL; *n* = 137, 64.1% in PL). Similar differences emerged for dream vividness (*Χ* ^2^_3_ = 35.35, *p* < .001). Again, the proportion of participants who reported a decrease in this measure was stable (*n* = 31, 14.5% in TL; *n* = 29, 13.6% in PL), whereas increases were more seldom reported in PL than in TL (*n* = 86, 40.2% in TL; *n* = 38, 17.8% in PL) and reporting no change was more frequent in PL (*n* = 97, 45.3% in TL; *n* = 147, 68.7% in PL).

Finally, using McNemar's test, we found no difference (*Χ* ^2^_1_ = 1.10, *p* = .292) in the proportion of participants reporting Covid‐19‐related dreams between TL (*n* = 64; 29.91%) and PL (*n* = 55; 25.70%).

### 3.6 Predictors of changes in dream frequency, length and vividness during TL and PL

Tables S1–S6 display the results of the multinomial logistic regressions on changes, during TL (*n* = 1622) and PL (*n* = 214), in dream frequency, length and vividness, respectively.

During TL, decreased dream frequency was significantly predicted by younger age (*b* = −0.02, OR = 0.97; 95% CI = 0.96–0.98), female gender (*b* = 0.33, OR = 1.38; 95% CI = 1.01–1.89) and worsened sleep quality (*b* = 0.07, OR = 1.07; 95% CI = 1.03–1.11), whereas increased dream frequency was predicted by younger age (*b* = −0.02, OR = 0.98; 95% CI = 0.97–0.99) and increased negative dream emotionality (*b* = 0.44, OR = 1.55; 95% CI = 1.35–1.78). As for PL, decreased dream frequency was predicted by younger age (*b* = −0.04, OR = 0.96; 95% CI = 0.92–0.99), very positive mood (*b* = −12.21, OR = 0.01; 95% CI = 0.01–0.01) and increased negative dream emotionality (*b* = 0.70, OR = 2.01; 95% CI = 1.15–3.50). Instead, only increased negative dream emotionality (*b* = 0.83, OR: 2.30; 95% CI = 1.46–3.64) was associated with increased dream frequency.

Decreased dream length during TL was associated with younger age (*b* = −0.03, OR = 0.97; 95% CI = 0.95–0.98) and worsened sleep quality (*b* = 0.07, OR = 1.07; 95% CI = 1.03–1.12). As for increased dream length in the same period (TL), it was predicted by younger age (*b* = −0.02, OR = 0.98; 95% CI = 0.97–0.99), delayed sleep midpoint (*b* = 0.12, OR = 1.13; 95% CI = 1.03–1.24) and increased negative dream emotionality (*b* = 0.23, OR = 1.26; 95% CI = 1.11–1.44). During PL, decreased dream length was associated with younger age (*b* = −0.05, OR = 0.95; 95% CI = 0.91–0.99) and increased negative dream emotionality (*b* = 0.63, OR = 1.87; 95% CI = 1.06–3.31), whereas only increased negative dream emotionality emerged as a significant predictor of increased dream length (*b* = 0.45, OR = 1.57; 95% CI = 1.00–2.48).

Decreased dream vividness during TL was associated with younger age (*b* = −0.02, OR = 0.98; 95% CI = 0.97–0.99), female gender (*b* = 0.42, OR = 1.52; 95% CI = 1.08–2.13), very negative mood (*b* = 1.17, OR = 3.24; 95% CI = 1.06–9.93), worsened sleep quality (*b* = 0.06, OR = 1.06; 95% CI = 1.02–1.10) and increased negative dream emotionality (*b* = 0.16, OR = 1.18; 95% CI = 1.00–1.39). In the same period (TL), decreased dream vividness was predicted by younger age (*b* = −0.01, OR = 0.99; 95% CI = 0.98–1.00), female gender (*b* = 0.47, OR = 1.60; 95% CI = 1.22–2.10), moderate stress (*b* = 0.40, OR = 1.50; 95% CI = 1.07–2.09) and increased negative dream emotionality (*b* = 0.42, OR = 1.53; 95% CI = 1.34–1.75). As for PL, no significant predictors emerged for decreased dream vividness, whereas increased dream vividness was negatively associated with very positive mood (*b* = −12.70, OR = 0.01; 95% CI = 0.01–0.01).

### 3.7 Predictors of changes in dream emotional valence during TL and PL

The increase in negative dream emotionality from pre‐TL to TL (*F*_8,1613_ = 29.81, *p* < .001, Adj.*R* ^2^ = 0.124; Table S7) was predicted by female gender (*β* = 0.104, *t* = 2.062, *p* = .040), higher stress (*β* = 0.113, *t* = 2.57, *p* = .010), more negative mood (*β* = 0.052, *t* = 2.91, *p* = .004) and worsened sleep quality (*β* = 0.069, *t* = 10.79, *p* < .001).

As for PL (*F*_8,205_ = 5.536, *p* < .001, Adj.*R* ^2^ = 0.146; Table S8), worsened sleep quality was the only significant predictor (*β* = 0.098, *t* = 5.246, *p* < .001). In addition, a trend toward significance emerged for stress, with higher stress associated with a more pronounced shift toward negative dream affect (*β* = 0.235, *t* = 1.778, *p* = .077).

### 3.8 Predictors of occurrence of Covid‐19‐related dreams during TL and PL

During TL (Table S9), the presence of Covid‐19‐related dreams was predicted by female gender (*b* = 0.379, OR = 1.46; 95% CI = 1.09–1.96), higher general fear (*b* = 0.379, OR = 1.46; 95% CI = 1.13–1.89), increased dream vividness (*b* = 0.443, OR = 1.56; 95% CI = 1.14–2.16) and increased negative dream affect (*b* = 0.348, OR = 1.42; 95% CI = 1.24–1.62).

As for PL (Table S10), the only predictor was higher general fear (*b* = 1.057, OR = 2.88; 95% CI = 1.26–6.60).

## 4 DISCUSSION

This study addresses the effects of the first and second waves of the Covid‐19 pandemic on dream features in an Italian sample. Specifically, we present data from a survey administered to a large sample (1,622 participants) during the first wave of the pandemic and related TL (spring 2020), as well as data from a follow‐up survey administered to a sub‐group of the same original sample (214 participants) in autumn 2020, when Italy underwent a resurgence of Covid‐19 cases (reaching a higher rate than that attained in spring) and the government responded with a second, PL.

A first important result is the proportion of subjects reporting changes in dream features in both lockdowns. About half of the sample showed changes in dream frequency, length, vividness and emotional valence during the first lockdown relative to the preceding period. Our longitudinal analysis on the 214 participants who responded to the follow‐up survey showed that significantly fewer participants reported similar changes (in dream frequency, length and vividness) during the second, PL relative to the first, suggesting some sort of adaptation. Also, this difference is coherent with the lower impact of the second lockdown on general lifestyle and habits due to the looser restrictions. This considered, the proportion of participants displaying changes in the second lockdown relative to the previous period remains impressive, exceeding 40% for dream frequency, and 30% for dream length and vividness.

These data are in line with results from previous surveys reporting significant changes in dream features in the general population during the first wave of the pandemic (Barrett, 2020; Gorgoni et al., 2021; MacKay & DeCicco, 2020; Mota et al., 2020; Schredl & Bulkeley, 2020; Wang, Zemmelman, Hong, Feng, & Shen, 2020; Xu et al., 2020). Specifically, while most studies focused on dream content, only two studies assessed changes in dream recall frequency compared with before the pandemic. Using the same question as the one used in our study, Schredl and Bulkeley (2020) found a proportion of subjects reporting increased dream frequency (29%) very similar to the one we observed during the same time frame (30%) as well as during the autumn lockdown relative to the preceding month (again, 30%). The other study (Gorgoni et al., 2021), instead, used retrospective questions. The authors found a significant increase of dream recall frequency during the first lockdown, as well as an increase of perceived length and vividness of dreams. To our knowledge, no other study has investigated dream length and vividness during the Covid‐19 pandemic.

It is worth noting that, during both lockdowns, all four variables (frequency, length, vividness and negative emotionality) were more often increased than decreased compared with the preceding periods, in line with Gorgoni et al.’s findings (2021) on the first pandemic wave. To this regard, results from our regression analyses suggest a possible role of sleep quality in mediating the effects of the stressful situation on the process of dream recall. Indeed, aside from its association with dream emotionality in both lockdowns (a finding that will be discussed later on), we found that worsened sleep quality significantly predicted decreases but not increases in dream frequency, length and vividness, at least in TL. This finding is in contrast to previous data supporting an association of poor sleep quality (especially frequent nocturnal awakenings) with increased dream recall frequency (Schredl, Schäfer, Weber, & Heuser, 1998; van Wyk, Solms, & Lipinska, 2019). However, the existence and direction of this relationship are not yet well established, as other studies have shown either none (Seong, Jung, Park, Choi, & Joo, 2017) or opposite associations (Brand et al., 2011; Pagel & Shocknesse, 2007). Nevertheless, as also suggested by the different results obtained in healthy and sleep‐disordered populations (Schredl, 2001), it cannot be excluded that the relationships between sleep quality and dream recall are modulated, in turn, by the degree of sleep impairment, which has not been considered in our study. Interestingly, instead, the delayed sleep timing (here expressed by sleep midpoint) does not appear to affect dream features, as shown by the absence of significant associations with most dream measures.

As for prevalent emotional valence of dreams, we found significant increases in negative emotionality during both lockdowns compared with the periods before them, in line with several previous studies conducted during the first pandemic wave (Barrett, 2020; Mota et al., 2020; Schredl & Bulkeley, 2020; Wang et al., 2020; Xu et al., 2020). This profile was confirmed by our longitudinal analysis, showing a significant oscillation of dream valence across the four time‐points in the 214 respondents of the follow‐up survey: negative dream affect increased during TL, then decreased in pre‐PL (when the lockdown was interrupted), and newly increased in PL. The fact that, between the two lockdowns, negative emotional valence returned almost to baseline level (i.e. the pre‐pandemic period) suggests that dream emotionality is closely related to lifestyle and emotional changes of daily life. Results from our regression analyses give hints to the main determinants of the negative emotional shifts that occurred during both lockdowns. The increase of negative dream emotionality was predicted, during the first lockdown, by stress, negative mood, female gender and worsening of sleep quality, in line with data from a few previous studies conducted during the first pandemic wave. Specifically, Schredl and Bulkeley (2020) and Barrett (2020) reported greater increases of dream negative affect in women. Also, the proportion of negative versus positive dream emotions was found to be significantly higher in females, poor sleepers, younger subjects, subjects with PTSD‐related symptoms, depression and anxiety symptoms (Gorgoni et al., 2021). Finally, Scarpelli et al., (2021) found associations of higher nightmare frequency with female gender, wake after sleep onset time, sleep problems, anxiety and depression, among other variables. Interestingly, we found that increased negative dream affect during the second lockdown was predicted, instead, only by the impoverishment of sleep quality and by stress, though the latter failed to reach significance. This finding appears in contrast with our results on the participants’ general state of worry during the second lockdown. In fact, as hypothesized, the increase in the number of daily Covid‐19 cases in autumn 2020 relative to the first pandemic wave was reflected in a general increase of scores in psychological measures (negative mood, fear and stress), which was coherent with the strikingly higher percentage of participants who reported knowing someone who was positive to Covid‐19 at the follow‐up survey. This higher psychological distress was also confirmed by our longitudinal analysis (on 214 participants) showing significantly higher general fear and fear of contagion in the second relative to the first lockdown. The lack of associations between dream emotionality and psychological variables in PL suggests that the negative emotional shift observed in dreams was unrelated to this worsening of waking emotionality. However, it must be acknowledged that the precise relationships between waking and dream emotionality are still not fully understood (Conte et al., 2020, 2021), especially regarding the mechanisms through which positive and negative affects of the waking state influence the corresponding emotional dimensions of the dream. Indeed, recent data from our group (Conte et al., 2020, 2021) call into question the existence of a direct reflection of prevalent emotional valence of wake into that of the dream. Furthermore, the possibility should be considered that the quality of psychological distress differs between the two pandemic waves, with that experienced during the first wave being more acute and that characterizing the second wave being more chronic. This could, in turn, bear differential effects on sleep mentation. Anyhow, our findings confirm the important modulating role played by sleep quality in determining dream emotional valence (already highlighted in Conte et al., 2021).

Concerning prevalent dream affect, another interesting finding is that the shift to more negative dream emotionality appeared, both in TL and PL, as a significant predictor of changes in dream frequency, length and vividness in most regression analyses. Specifically, it predicted both increases and decreases of the other dream variables (though in two cases there was only a trend). This pattern suggests that the emotional valence of dreams could be a particularly relevant factor in determining several aspects of dream recall (both its frequency and the perceived characteristics of recalled dreams), possibly through a “salience effect”. Indeed, the salience hypothesis of dream recall suggests that the subjective relevance of dream contents significantly affects their recall (Cohen & MacNeilage, 1974). Therefore, it is plausible that, given a higher salience of negatively toned dreams, their other quantitative and qualitative aspects are influenced in different directions according to individual characteristics (for a review on individual differences affecting dream features, see Blagrove & Pace‐Schott, 2010). For instance, while negatively toned (i.e. more salient) dreams could be perceived as longer and more vivid by most subjects, they could be recalled as shorter and less vivid by others due to some sort of cognitive suppression mechanism. Also, state factors, such as pre‐sleep mood, sleep fragmentation, interference upon awakening or physiological arousal during the dream could modulate this mechanism (Blagrove & Pace‐Schott, 2010; Cohen, 1974).

Our pattern of findings also confirmed the wide literature on the modulating role of age on dream variables (Giambra, Jung, & Grodsky, 1996; Nielsen, 2012; Schredl, Erlacher, Reiner, & Woll, 2014). Younger age predicted both increases and decreases in dream frequency, length and vividness during the first lockdown. In analogy with that proposed for emotional valence, this finding could be explained recurring to the different salience of dreams at different ages. Indeed, while it has been repeatedly reported that dream recall decreases with age (Giambra et al., 1996; Nielsen, 2012; Schredl et al., 2014), this age‐related decline has been ascribed by several authors to a diminished interest in dreams (Giambra et al., 1996; Strunz, 1993). Following this line of reasoning, it is plausible that younger subjects of our sample, due to the greater attention paid to dreaming, were more prone to notice changes in their oneiric activity determined by the pandemic, irrespective of the direction of these changes.

As for gender differences, our data suggest that females are more affected by the pandemic‐related changes in sleep mentation, in line with several other studies from the first pandemic wave reporting that women showed greater consequences than men, both on sleep quality (Casagrande et al., 2020; Cellini et al., 2021; Franceschini et al., 2020) and dream measures (Barrett, 2020; Gorgoni et al., 2021; Pesonen et al., 2020; Scarpelli et al., 2021; Schredl & Bulkeley, 2020).

Finally, we observed, during both lockdowns, a very similar proportion of subjects reporting COVID‐19‐related dreams (25%), and reporting to face, in these dreams, problematic situations resembling those of their waking life. The proportion was quite different from that reported by other studies conducted in the USA (8.15%; Schredl & Bulkeley, 2020) and Finland (55%; Pesonen et al., 2020), while it was similar to that reported by another Italian study (20%; Iorio et al., 2020), suggesting a role of cultural differences in modulating dream imagery and the incorporation of waking life events in dreams (incorporation continuity hypothesis; Schredl, 2012; Schredl & Hofmann, 2003), although differences in data collection methods should also be taken into account. Also, Covid‐19‐related dream content was more probable in subjects showing a more pronounced shift to negative dream emotionality, in line with the findings of Schredl and Bulkeley, (2020) and, unsurprisingly, in women and in participants reporting higher general fear and increased dream vividness.

A few limitations should be taken into account in the interpretation of our results. First, participants self‐selected and thus may have already had changes in their sleep and dreaming features that prompted their interest in completing the survey, thereby limiting generalizability of our data to the population at large. Second, retrospective assessments on the pre‐lockdown periods may have resulted in recall bias. Additionally, assumptions about the impact of the crisis (as well as lifted restrictions) on sleep and dreaming may have led participants to overestimate the changes in their sleep and oneiric activity in the direction that would be expected based on confirmation bias. Despite these limitations, which are common to most dream studies conducted during the COVID‐19 pandemic, these findings provide chronological depth to the existing literature on dreaming and its sensitivity to daytime emotionality and lifestyle changes during a public health crisis.

In conclusion, in line with previous research conducted in several countries during the first wave of the Covid‐19 pandemic, our study confirms that dream activity is significantly affected by major life changes such as those imposed by a worldwide health emergency. In fact, we show that changes in dream features (namely prevalent emotional tone, recall frequency, perceived length and vividness) brought about by the pandemic involve a very high proportion of subjects (about half of the sample during the first pandemic wave and slightly less during the second). More importantly, to our knowledge, this is the first study to follow the evolution of these changes over the course of a pandemic, taking into account both the first, more acute phase, and related strong restrictions, and a second phase, characterized by lower restrictions in the face of prolonged stress. In this sense, our findings highlight that dream features are extremely sensible to lifestyle and wake‐time emotional changes, in that their modifications closely followed the oscillations in the course of the pandemic and of its consequences on everyday life. In other words, other than undergoing some sort of habituation effect over time, dreaming activity in a large part of the population appears to have been significantly shaped by current public events in their development over time. In this perspective, our findings add support to the idea, proposed by several authors (Schredl & Bulkeley, 2020), that dream features and their changes may be considered as a useful index of psychological impact in emergency situations.

## CONFLICT OF INTEREST

The authors declare no financial conflicts of interest, and any personal or financial support and involvement with organizations with financial interest in the subject matter of the paper.

## AUTHOR CONTRIBUTIONS

All authors contributed in a meaningful way to this manuscript. Conceptualization, F.C., F.G. and G.F.; methodology, F.C., N.C. and G.F.; formal analysis, N.C. and O.D.; investigation, O.D., A.C., M.C. and S.M.; writing—original draft preparation, F.C., M.L.R and O.D.; writing—review and editing, F.C., M.L.R., N.C. and G.F.; visualization, F.C. and N.C.; supervision, F.G. and G.F.; project administration, F.C., F.G. and G.F. All authors have read and agreed to the published version of the manuscript.

## Supporting information

Tables S1‐S10

## Data Availability

The data that support the findings of this study are available on request from the corresponding author. The data are not publicly available due to privacy or ethical restrictions.
