# Supplementary material for: Changes in dream features across the first and second waves of the Covid‐19 pandemic
Source: J Sleep Res. 2021 Jun 22;31(1):e13425. doi: 10.1111/jsr.13425 (PMC8420319; doi:10.1111/jsr.13425)
Supplement: Supplementary file 1 — Tables S1‐S10 [file JSR-31-0-s001.docx]

**SUPPLEMENTARY MATERIAL**

**Table S1.** Multinomial logistic regression analysis on Dream Frequency during the Total Lockdown

|  | | ***Decreased (Reference = Equal)*** | | | | | ***Increased (Reference = Equal)*** | | | | |
| --- | --- | --- | --- | --- | --- | --- | --- | --- | --- | --- | --- |
| ***Variable*** | | ***b*** | ***p*** | ***OR*** | ***95%CI-*** | ***95%CI+*** | ***b*** | ***p*** | ***OR*** | ***95%CI-*** | ***95%CI+*** |
| **Age** |  | -0.025 | **<.001** | 0.975 | 0.965 | 0.985 | -0.016 | **<.001** | 0.984 | 0.975 | 0.993 |
| **Gender** | |  |  |  |  |  |  |  |  |  |  |
|  | *Female* | 0.326 | **.042** | 1.385 | 1.012 | 1.894 | 0.217 | .117 | 1.243 | 0.947 | 1.631 |
|  | *Male* | Reference |  |  |  |  | Reference |  |  |  |  |
| **Mood** | |  |  |  |  |  |  |  |  |  |  |
|  | *Very Positive* | 0.613 | .144 | 1.845 | 0.811 | 4.199 | -0.088 | .778 | 0.916 | 0.497 | 1.688 |
|  | *Moderately Positive* | 0.460 | .272 | 1.584 | 0.697 | 3.598 | -0.088 | .777 | 0.916 | 0.501 | 1.676 |
|  | *Neutral* | Reference |  |  |  |  | Reference |  |  |  |  |
|  | *Moderately Negative* | 0.782 | .066 | 2.185 | 0.949 | 5.028 | 0.152 | .633 | 1.164 | 0.624 | 2.17 |
|  | *Very Negative* | 0.778 | .141 | 2.178 | 0.773 | 6.139 | 0.424 | .325 | 1.528 | 0.657 | 3.551 |
| **Stress** | |  |  |  |  |  |  |  |  |  |  |
|  | *Neutral* | Reference |  |  |  |  | Reference |  |  |  |  |
|  | *Moderately Stressed* | 0.125 | .525 | 1.133 | 0.771 | 1.664 | 0.261 | .128 | 1.298 | 0.928 | 1.816 |
|  | *Extremely Stressed* | 0.124 | .657 | 1.132 | 0.654 | 1.960 | 0.044 | .864 | 1.045 | 0.630 | 1.735 |
| **General Fear** | |  |  |  |  |  |  |  |  |  |  |
|  | *Neutral* | Reference |  |  |  |  |  |  |  |  |  |
|  | *Moderately Afraid* | -0.135 | .469 | 0.874 | 0.607 | 1.259 | 0.029 | .861 | 1.029 | 0.745 | 1.423 |
|  | *Extremely Afraid* | 0.247 | .390 | 1.281 | 0.728 | 2.251 | -0.039 | .887 | 0.962 | 0.562 | 1.646 |
| **Fear of contagion** | |  |  |  |  |  |  |  |  |  |  |
|  | *Neutral* | Reference |  |  |  |  |  |  |  |  |  |
|  | *Moderately Afraid* | 0.103 | .711 | 1.108 | 0.643 | 1.911 | 0.031 | .897 | 1.032 | 0.645 | 1.65 |
|  | *Extremely Afraid* | 0.300 | .315 | 1.349 | 0.752 | 2.420 | 0.001 | .999 | 1.00 | 0.601 | 1.666 |
| **Δ PSQI** | | 0.067 | **<.001** | 1.069 | 1.028 | 1.112 | 0.032 | .081 | 1.033 | 0.996 | 1.071 |
| **Δ Sleep Midpoint** | | 0.056 | .292 | 1.058 | 0.953 | 1.175 | 0.091 | .062 | 1.096 | 0.995 | 1.206 |
| **Δ Dream Emotional Valence** | | 0.136 | .077 | 1.145 | 0.986 | 1.331 | 0.438 | **<.001** | 1.549 | 1.349 | 1.779 |
| **Overal model test:** χ2_30_=166.56, p<.001, R^2^_N_=.067.  *Notes:* n=1622; PSQI: Pittsburgh Sleep Quality Index; R^2^_N_: Nagelkerke’s R^2^ | | | | | | | | | | | |

**Table S2.** Multinomial logistic regression analysis on Dream Frequency during the Partial Lockdown

|  | | ***Decreased (Reference = Equal)*** | | | | | ***Increased (Reference = Equal)*** | | | | |
| --- | --- | --- | --- | --- | --- | --- | --- | --- | --- | --- | --- |
| ***Variable*** | | ***b*** | ***p*** | ***OR*** | ***95%CI-*** | ***95%CI+*** | ***b*** | ***p*** | ***OR*** | ***95%CI-*** | ***95%CI+*** |
| **Age** |  | -0.040 | **.044** | 0.961 | 0.924 | 0.999 | -0.006 | .663 | 0.994 | 0.970 | 1.020 |
| **Gender** | |  |  |  |  |  |  |  |  |  |  |
|  | *Female* | -0.148 | .777 | 0.862 | 0.31 | 2.400 | 0.340 | .413 | 1.404 | 0.622 | 3.169 |
|  | *Male* | Reference |  |  |  |  | Reference |  |  |  |  |
| **Mood** | |  |  |  |  |  |  |  |  |  |  |
|  | *Very Positive* | -12.209 | **<.001** | 0.001 | 0.001 | 0.001 | -1.023 | .462 | 0.360 | 0.023 | 5.503 |
|  | *Moderately Positive* | -0.062 | .926 | 0.940 | 0.253 | 3.494 | -0.032 | .942 | 0.968 | 0.405 | 2.314 |
|  | *Neutral* | Reference |  |  |  |  | Reference |  |  |  |  |
|  | *Moderately Negative* | 0.436 | .462 | 1.547 | 0.483 | 4.951 | -0.381 | .406 | 0.683 | 0.278 | 1.678 |
|  | *Very Negative* | -0.659 | .525 | 0.517 | 0.068 | 3.940 | -1.072 | .188 | 0.342 | 0.070 | 1.687 |
| **Stress** | |  |  |  |  |  |  |  |  |  |  |
|  | *Neutral* | Reference |  |  |  |  | Reference |  |  |  |  |
|  | *Moderately Stressed* | 0.887 | .439 | 2.428 | 0.257 | 22.958 | -0.837 | .133 | 0.433 | 0.145 | 1.291 |
|  | *Extremely Stressed* | 1.129 | .390 | 3.092 | 0.235 | 40.661 | -0.842 | .285 | 0.431 | 0.092 | 2.018 |
| **General Fear** | |  |  |  |  |  |  |  |  |  |  |
|  | *Neutral* | Reference |  |  |  |  |  |  |  |  |  |
|  | *Moderately Afraid* | -0.553 | .351 | 0.575 | 0.180 | 1.839 | 0.001 | .998 | 1.001 | 0.377 | 2.662 |
|  | *Extremely Afraid* | -0.517 | .641 | 0.596 | 0.068 | 5.232 | 0.368 | .647 | 1.445 | 0.300 | 6.969 |
| **Fear of contagion** | |  |  |  |  |  |  |  |  |  |  |
|  | *Neutral* | Reference |  |  |  |  |  |  |  |  |  |
|  | *Moderately Afraid* | 0.225 | .858 | 1.253 | 0.107 | 14.667 | -0.237 | .809 | 0.789 | 0.115 | 5.418 |
|  | *Extremely Afraid* | -0.105 | .938 | 0.901 | 0.065 | 12.488 | 0.006 | .995 | 1.006 | 0.136 | 7.461 |
| **Δ PSQI** | | 0.022 | .779 | 1.022 | 0.876 | 1.193 | 0.065 | .284 | 1.067 | 0.947 | 1.202 |
| **Δ Sleep Midpoint** | | -0.420 | .078 | 0.657 | 0.412 | 1.049 | -0.031 | .869 | 0.969 | 0.670 | 1.402 |
| **Δ Dream Emotional Valence** | | 0.698 | **.014** | 2.010 | 1.155 | 3.498 | 0.835 | **<.001** | 2.305 | 1.460 | 3.638 |
| **Overal model test:** χ2_30_=47.42, p=.023, R^2^_N_=.151.  *Notes:* n=214; PSQI: Pittsburgh Sleep Quality Index; R^2^_N_: Nagelkerke’s R^2^ | | | | | | | | | | | |

**Table S3.** Multinomial logistic regression analysis on Dream Length during the Total Lockdown

|  | | ***Decreased (Reference = Equal)*** | | | | | ***Increased (Reference = Equal)*** | | | | |
| --- | --- | --- | --- | --- | --- | --- | --- | --- | --- | --- | --- |
| ***Variable*** | | ***b*** | ***p*** | ***OR*** | ***95%CI-*** | ***95%CI+*** | ***b*** | ***p*** | ***OR*** | ***95%CI-*** | ***95%CI+*** |
| **Age** |  | -0.034 | **<.001** | 0.967 | 0.955 | 0.979 | -0.019 | **<.001** | 0.981 | 0.972 | 0.990 |
| **Gender** | |  |  |  |  |  |  |  |  |  |  |
|  | *Female* | 0.220 | .220 | 1.246 | 0.877 | 1.771 | 0.199 | .154 | 1.221 | 0.928 | 1.605 |
|  | *Male* | Reference |  |  |  |  | Reference |  |  |  |  |
| **Mood** | |  |  |  |  |  |  |  |  |  |  |
|  | *Very Positive* | -0.058 | .892 | 0.944 | 0.407 | 2.187 | -0.108 | .740 | 0.897 | 0.474 | 1.700 |
|  | *Moderately Positive* | 0.174 | .682 | 1.190 | 0.517 | 2.740 | 0.120 | .709 | 1.127 | 0.600 | 2.118 |
|  | *Neutral* | Reference |  |  |  |  | Reference |  |  |  |  |
|  | *Moderately Negative* | 0.363 | .398 | 1.438 | 0.619 | 3.343 | 0.245 | .459 | 1.278 | 0.668 | 2.442 |
|  | *Very Negative* | 0.049 | .927 | 1.050 | 0.367 | 3.002 | 0.152 | .727 | 1.164 | 0.497 | 2.727 |
| **Stress** | |  |  |  |  |  |  |  |  |  |  |
|  | *Neutral* | Reference |  |  |  |  | Reference |  |  |  |  |
|  | *Moderately Stressed* | 0.203 | .378 | 1.225 | 0.780 | 1.926 | 0.201 | .241 | 1.223 | 0.873 | 1.712 |
|  | *Extremely Stressed* | 0.322 | .298 | 1.381 | 0.752 | 2.535 | 0.140 | .583 | 1.150 | 0.698 | 1.897 |
| **General Fear** | |  |  |  |  |  |  |  |  |  |  |
|  | *Neutral* | Reference |  |  |  |  |  |  |  |  |  |
|  | *Moderately Afraid* | -0.039 | .855 | 0.962 | 0.636 | 1.455 | -0.007 | .968 | 0.993 | 0.721 | 1.369 |
|  | *Extremely Afraid* | 0.481 | .122 | 1.617 | 0.879 | 2.976 | 0.243 | .365 | 1.275 | 0.754 | 2.156 |
| **Fear of contagion** | |  |  |  |  |  |  |  |  |  |  |
|  | *Neutral* | Reference |  |  |  |  |  |  |  |  |  |
|  | *Moderately Afraid* | 0.160 | .617 | 1.173 | 0.627 | 2.196 | 0.012 | .960 | 1.012 | 0.639 | 1.604 |
|  | *Extremely Afraid* | 0.279 | .410 | 1.322 | 0.680 | 2.567 | -0.250 | .338 | 0.782 | 0.473 | 1.293 |
| **Δ PSQI** | | 0.071 | **.001** | 1.074 | 1.029 | 1.120 | 0.024 | .183 | 1.025 | 0.989 | 1.062 |
| **Δ Sleep Midpoint** | | 0.008 | .888 | 1.008 | 0.899 | 1.131 | 0.124 | **.010** | 1.132 | 1.030 | 1.245 |
| **Δ Dream Emotional Valence** | | 0.121 | .135 | 1.129 | 0.963 | 1.324 | 0.235 | **<.001** | 1.264 | 1.106 | 1.445 |
| **Overal model test:** χ2_30_=138.87, p<.001, R^2^_N_=.059.  *Notes:* n=1622; PSQI: Pittsburgh Sleep Quality Index; R^2^_N_: Nagelkerke’s R^2^ | | | | | | | | | | | |

**Table S4.** Multinomial logistic regression analysis on Dream Length during the Partial Lockdown

|  | | ***Decreased (Reference = Equal)*** | | | | | ***Increased (Reference = Equal)*** | | | | |
| --- | --- | --- | --- | --- | --- | --- | --- | --- | --- | --- | --- |
| ***Variable*** | | ***b*** | ***p*** | ***OR*** | ***95%CI-*** | ***95%CI+*** | ***b*** | ***p*** | ***OR*** | ***95%CI-*** | ***95%CI+*** |
| **Age** |  | -0.048 | **.027** | 0.953 | 0.913 | 0.994 | -0.009 | .519 | 0.991 | 0.965 | 1.018 |
| **Gender** | |  |  |  |  |  |  |  |  |  |  |
|  | *Female* | -0.444 | .422 | 0.641 | 0.217 | 1.896 | -0.312 | .456 | 0.732 | 0.322 | 1.662 |
|  | *Male* | Reference |  |  |  |  | Reference |  |  |  |  |
| **Mood** | |  |  |  |  |  |  |  |  |  |  |
|  | *Very Positive* | 2.195 | .154 | 8.981 | 0.438 | 184.028 | 0.554 | .692 | 1.741 | 0.113 | 26.898 |
|  | *Moderately Positive* | 0.250 | .719 | 1.283 | 0.330 | 4.996 | -0.277 | .589 | 0.758 | 0.277 | 2.071 |
|  | *Neutral* | Reference |  |  |  |  | Reference |  |  |  |  |
|  | *Moderately Negative* | 0.144 | .832 | 1.155 | 0.304 | 4.390 | 0.340 | .470 | 1.405 | 0.558 | 3.536 |
|  | *Very Negative* | -0.744 | .473 | 0.475 | 0.062 | 3.621 | -1.109 | .213 | 0.330 | 0.058 | 1.888 |
| **Stress** | |  |  |  |  |  |  |  |  |  |  |
|  | *Neutral* | Reference |  |  |  |  | Reference |  |  |  |  |
|  | *Moderately Stressed* | 0.240 | .778 | 1.271 | 0.240 | 6.735 | 0.425 | .528 | 1.529 | 0.409 | 5.716 |
|  | *Extremely Stressed* | 1.927 | .069 | 6.868 | 0.858 | 55.002 | 1.532 | .086 | 4.627 | 0.804 | 26.624 |
| **General Fear** | |  |  |  |  |  |  |  |  |  |  |
|  | *Neutral* | Reference |  |  |  |  |  |  |  |  |  |
|  | *Moderately Afraid* | -1.041 | .084 | 0.353 | 0.108 | 1.152 | -0.524 | .282 | 0.592 | 0.228 | 1.538 |
|  | *Extremely Afraid* | -0.212 | .832 | 0.809 | 0.114 | 5.744 | -0.241 | .785 | 0.786 | 0.139 | 4.445 |
| **Fear of contagion** | |  |  |  |  |  |  |  |  |  |  |
|  | *Neutral* | Reference |  |  |  |  |  |  |  |  |  |
|  | *Moderately Afraid* | -1.188 | .268 | 0.305 | 0.037 | 2.496 | -0.202 | .851 | 0.817 | 0.100 | 6.698 |
|  | *Extremely Afraid* | -1.642 | .160 | 0.194 | 0.020 | 1.914 | -1.000 | .380 | 0.368 | 0.039 | 3.436 |
| **Δ PSQI** | | -0.142 | .066 | 0.867 | 0.745 | 1.009 | -0.001 | .991 | 0.999 | 0.879 | 1.136 |
| **Δ Sleep Midpoint** | | 0.276 | .237 | 1.318 | 0.834 | 2.081 | 0.340 | .098 | 1.405 | 0.939 | 2.103 |
| **Δ Dream Emotional Valence** | | 0.626 | **.031** | 1.870 | 1.057 | 3.308 | 0.452 | **.050** | 1.571 | 0.996 | 2.476 |
| **Overal model test:** χ2_30_=48.42, p=.018, R^2^_N_=.163.  *Notes:* n=214; PSQI: Pittsburgh Sleep Quality Index; R^2^_N_: Nagelkerke’s R^2^ | | | | | | | | | | | |

**Table S5.** Multinomial logistic regression analysis on Dream Vividness during the Total Lockdown

|  | | ***Decreased (Reference = Equal)*** | | | | | ***Increased (Reference = Equal)*** | | | | |
| --- | --- | --- | --- | --- | --- | --- | --- | --- | --- | --- | --- |
| ***Variable*** | | ***b*** | ***p*** | ***OR*** | ***95%CI-*** | ***95%CI+*** | ***b*** | ***p*** | ***OR*** | ***95%CI-*** | ***95%CI+*** |
| **Age** |  | -0.017 | **.003** | 0.983 | 0.972 | 0.994 | -0.009 | **.041** | 0.991 | 0.982 | 1.000 |
| **Gender** | |  |  |  |  |  |  |  |  |  |  |
|  | *Female* | 0.419 | **.015** | 1.520 | 1.084 | 2.132 | 0.473 | **<.001** | 1.605 | 1.225 | 2.103 |
|  | *Male* | Reference |  |  |  |  | Reference |  |  |  |  |
| **Mood** | |  |  |  |  |  |  |  |  |  |  |
|  | *Very Positive* | 0.460 | .327 | 1.584 | 0.631 | 3.978 | -0.183 | .547 | 0.833 | 0.460 | 1.510 |
|  | *Moderately Positive* | 0.363 | .438 | 1.438 | 0.574 | 3.600 | -0.228 | .448 | 0.796 | 0.442 | 1.434 |
|  | *Neutral* | Reference |  |  |  |  | Reference |  |  |  |  |
|  | *Moderately Negative* | 0.745 | .116 | 2.106 | 0.832 | 5.327 | -0.091 | .769 | 0.913 | 0.497 | 1.676 |
|  | *Very Negative* | 1.175 | **.040** | 3.237 | 1.055 | 9.934 | 0.404 | .335 | 1.498 | 0.659 | 3.406 |
| **Stress** | |  |  |  |  |  |  |  |  |  |  |
|  | *Neutral* | Reference |  |  |  |  | Reference |  |  |  |  |
|  | *Moderately Stressed* | 0.203 | .345 | 1.225 | 0.804 | 1.865 | 0.402 | **.018** | 1.496 | 1.072 | 2.087 |
|  | *Extremely Stressed* | 0.036 | .906 | 1.037 | 0.570 | 1.887 | 0.333 | .184 | 1.395 | 0.854 | 2.279 |
| **General Fear** | |  |  |  |  |  |  |  |  |  |  |
|  | *Neutral* | Reference |  |  |  |  |  |  |  |  |  |
|  | *Moderately Afraid* | 0.087 | .671 | 1.091 | 0.729 | 1.633 | -0.043 | .790 | 0.958 | 0.700 | 1.312 |
|  | *Extremely Afraid* | 0.147 | .636 | 1.159 | 0.630 | 2.130 | -0.344 | .193 | 0.709 | 0.423 | 1.189 |
| **Fear of contagion** | |  |  |  |  |  |  |  |  |  |  |
|  | *Neutral* | Reference |  |  |  |  |  |  |  |  |  |
|  | *Moderately Afraid* | 0.039 | .900 | 1.039 | 0.569 | 1.897 | -0.180 | .438 | 0.835 | 0.53 | 1.316 |
|  | *Extremely Afraid* | 0.070 | .831 | 1.073 | 0.564 | 2.039 | -0.173 | .490 | 0.841 | 0.514 | 1.376 |
| **Δ PSQI** | | 0.058 | **.007** | 1.060 | 1.016 | 1.105 | 0.022 | .205 | 1.023 | 0.988 | 1.059 |
| **Δ Sleep Midpoint** | | -0.006 | .918 | 0.994 | 0.888 | 1.113 | 0.001 | .985 | 0.999 | 0.910 | 1.096 |
| **Δ Dream Emotional Valence** | | 0.163 | **.045** | 1.178 | 1.003 | 1.382 | 0.424 | **<.001** | 1.528 | 1.336 | 1.746 |
| **Overal model test:** χ2_30_=136.58, p<.001, R^2^_N_=.057.  *Notes:* n=1622; PSQI: Pittsburgh Sleep Quality Index; R^2^_N_: Nagelkerke’s R^2^ | | | | | | | | | | | |

**Table S6.** Multinomial logistic regression analysis on Dream Vividness during the Partial Lockdown

|  | | ***Decreased (Reference = Equal)*** | | | | | ***Increased (Reference = Equal)*** | | | | |
| --- | --- | --- | --- | --- | --- | --- | --- | --- | --- | --- | --- |
| ***Variable*** | | ***b*** | ***p*** | ***OR*** | ***95%CI-*** | ***95%CI+*** | ***b*** | ***p*** | ***OR*** | ***95%CI-*** | ***95%CI+*** |
| **Age** |  | -0.036 | .057 | 0.965 | 0.930 | 1.001 | -0.028 | .075 | 0.973 | 0.944 | 1.003 |
| **Gender** | |  |  |  |  |  |  |  |  |  |  |
|  | *Female* | -0.047 | .928 | 0.954 | 0.341 | 2.669 | -0.599 | .183 | 0.549 | 0.228 | 1.326 |
|  | *Male* | Reference |  |  |  |  | Reference |  |  |  |  |
| **Mood** | |  |  |  |  |  |  |  |  |  |  |
|  | *Very Positive* | 1.176 | .394 | 3.241 | 0.217 | 48.438 | -12.702 | **<.001** | 0.001 | 0.001 | 0.001 |
|  | *Moderately Positive* | -0.144 | .819 | 0.866 | 0.253 | 2.961 | -0.052 | .930 | 0.949 | 0.297 | 3.034 |
|  | *Neutral* | Reference |  |  |  |  | Reference |  |  |  |  |
|  | *Moderately Negative* | 0.314 | .593 | 1.368 | 0.433 | 4.326 | 0.898 | .084 | 2.454 | 0.885 | 6.806 |
|  | *Very Negative* | 0.047 | .959 | 1.048 | 0.177 | 6.214 | -0.045 | .959 | 0.956 | 0.171 | 5.355 |
| **Stress** | |  |  |  |  |  |  |  |  |  |  |
|  | *Neutral* | Reference |  |  |  |  | Reference |  |  |  |  |
|  | *Moderately Stressed* | -0.193 | .793 | 0.825 | 0.197 | 3.462 | 0.497 | .560 | 1.644 | 0.309 | 8.757 |
|  | *Extremely Stressed* | 0.464 | .633 | 1.59 | 0.237 | 10.679 | 1.047 | .305 | 2.849 | 0.386 | 21.024 |
| **General Fear** | |  |  |  |  |  |  |  |  |  |  |
|  | *Neutral* | Reference |  |  |  |  |  |  |  |  |  |
|  | *Moderately Afraid* | -0.718 | .182 | 0.488 | 0.170 | 1.401 | -0.101 | .856 | 0.904 | 0.302 | 2.700 |
|  | *Extremely Afraid* | -1.855 | .143 | 0.156 | 0.013 | 1.869 | -1.013 | .279 | 0.363 | 0.058 | 2.271 |
| **Fear of contagion** | |  |  |  |  |  |  |  |  |  |  |
|  | *Neutral* | Reference |  |  |  |  |  |  |  |  |  |
|  | *Moderately Afraid* | -0.415 | .689 | 0.66 | 0.087 | 5.027 | -0.069 | .948 | 0.933 | 0.115 | 7.573 |
|  | *Extremely Afraid* | -0.707 | .531 | 0.493 | 0.054 | 4.514 | -0.034 | .976 | 0.967 | 0.107 | 8.763 |
| **ΔPSQI** | | 0.099 | .217 | 1.104 | 0.944 | 1.290 | 0.047 | .473 | 1.048 | 0.922 | 1.192 |
| **Δ Sleep Midpoint** | | -0.158 | .497 | 0.854 | 0.540 | 1.348 | 0.144 | .478 | 1.155 | 0.776 | 1.718 |
| **Δ Dream Emotional Valence** | | 0.126 | .631 | 1.134 | 0.679 | 1.894 | 0.156 | .509 | 1.169 | 0.736 | 1.856 |
| **Overal model test:** χ2_30_=32.20, p=.358, R^2^_N_=.115.  *Notes:* n=214; PSQI: Pittsburgh Sleep Quality Index; R^2^_N_: Nagelkerke’s R^2^ | | | | | | | | | | | |

**Table S7.** Multiple linear regression analysis on changes in dream emotional valence during the Total Lockdown

| ***Variable*** | | ***F*** | ***β*** | ***t*** | ***p*** |
| --- | --- | --- | --- | --- | --- |
| **Age** | | 0.148 | -0.001 | -0.385 | .701 |
| **Gender** | |  |  |  |  |
|  | *Female* | 4.215 | 0.104 | 2.053 | **.040** |
|  | *Male* | Reference |  |  |  |
| **Mood** | | 8.525 | 0.052 | 2.920 | **.004** |
| **Stress** | | 6.602 | 0.113 | 2.569 | **.010** |
| **General Fear** | | 1.867 | 0.064 | 1.367 | .172 |
| **Fear of contagion** | | 1.356 | 0.036 | 1.165 | .244 |
| **Δ PSQI** | | 116.52 | 0.069 | 10.794 | **<.001** |
| **Δ Sleep Midpoint** | | 1.302 | 0.020 | 1.141 | .254 |
| **Overal model test:** F_8,1613_=29.81, p<.001, Adj.R^2^=.124.  *Notes:* n=1622; PSQI: Pittsburgh Sleep Quality Index | | | | |  |

**Table S8.** Multiple linear regression analysis on changes in dream emotional valence during the Partial Lockdown

| ***Variable*** | | ***F*** | ***β*** | ***t*** | ***p*** |
| --- | --- | --- | --- | --- | --- |
| **Age** | | 0.107 | -0.022 | -0.327 | .744 |
| **Gender** | |  |  |  |  |
|  | *Female* | 0.204 | 0.067 | 0.452 | .652 |
|  | *Male* | Reference |  |  |  |
| **Mood** | | 0.042 | -0.014 | -0.204 | .839 |
| **Stress** | | 3.161 | 0.127 | 1.778 | .077 |
| **General Fear** | | 0.108 | 0.023 | 0.329 | .742 |
| **Fear of contagion** | | 1.598 | 0.088 | 1.264 | .208 |
| **Δ PSQI** | | 27.518 | 0.344 | 5.246 | **<.001** |
| **Δ Sleep Midpoint** | | 0.745 | 0.057 | 0.863 | .389 |
| **Overal model test:** F_8,205_=5.536, p<.001, Adj.R^2^=.146.  *Notes:* n=214; PSQI: Pittsburgh Sleep Quality Index | | | | |  |

**Table S9.** Binary logistic regression analysis on the occurrence of Covid-19-related dreams during the Total Lockdown

| ***Variable*** | | ***b*** | ***p*** | ***OR*** | ***95%CI-*** | ***95%CI+*** |
| --- | --- | --- | --- | --- | --- | --- |
| **Age** |  | 0.001 | .852 | 1.001 | 0.992 | 1.010 |
| **Gender** | |  |  |  |  |  |
|  | *Female* | 0.379 | **.012** | 1.461 | 1.089 | 1.960 |
|  | *Male* | Reference |  |  |  |  |
| **Dream Frequency** | |  |  |  |  |  |
|  | *Increased* | 0.264 | .120 | 1.302 | 0.933 | 1.815 |
|  | *Equal* | Reference |  |  |  |  |
|  | *Decreased* | -0.223 | .274 | 0.800 | 0.537 | 1.193 |
| **Dream Length** | |  |  |  |  |  |
|  | *Increased* | 0.122 | .483 | 1.129 | 0.804 | 1.587 |
|  | *Equal* | Reference |  |  |  |  |
|  | *Decreased* | 0.158 | .477 | 1.172 | 0.757 | 1.812 |
| **Dream Vividness** | |  |  |  |  |  |
|  | *Increased* | 0.453 | **.006** | 1.572 | 1.142 | 2.165 |
|  | *Equal* | Reference |  |  |  |  |
|  | *Decreased* | 0.08 | .708 | 1.084 | 0.712 | 1.650 |
| **Mood** | | -0.044 | .369 | 0.957 | 0.868 | 1.054 |
| **Stress** | | 0.113 | .361 | 1.120 | 0.878 | 1.427 |
| **General Fear** | | 0.379 | **.004** | 1.461 | 1.131 | 1.887 |
| **Fear of contagion** | | 0.072 | .418 | 1.075 | 0.903 | 1.280 |
| **ΔPSQI** | | 0.003 | .875 | 1.003 | 0.969 | 1.038 |
| **Δ Sleep Midpoint** | | 0.042 | .388 | 1.043 | 0.948 | 1.148 |
| **Δ Emotional Dream Valence** | | 0.348 | **<.001** | 1.417 | 1.240 | 1.619 |
| **Overal model test:** χ2_15_=131.17, p<.001, R^2^_N_=.115.  *Notes:* n=1622; PSQI: Pittsburgh Sleep Quality Index; R^2^_N_: Nagelkerke’s R^2^ | | | | | | |

**Table S10.** Binary logistic regression analysis on the occurrence of Covid-19-related dreams during the Partial Lockdown

| ***Variable*** | | ***b*** | ***p*** | ***OR*** | ***95%CI-*** | ***95%CI+*** |
| --- | --- | --- | --- | --- | --- | --- |
| **Age** |  | -0.020 | .146 | 0.98 | 0.954 | 1.007 |
| **Gender** | |  |  |  |  |  |
|  | *Female* | 0.063 | .881 | 1.065 | 0.466 | 2.433 |
|  | *Male* | Reference |  |  |  |  |
| **Dream Frequency** | |  |  |  |  |  |
|  | *Increased* | -0.217 | .637 | 0.805 | 0.327 | 1.982 |
|  | *Equal* | Reference |  |  |  |  |
|  | *Decreased* | -0.606 | .308 | 0.546 | 0.17 | 1.748 |
| **Dream Length** | |  |  |  |  |  |
|  | *Increased* | -0.292 | .607 | 0.747 | 0.245 | 2.272 |
|  | *Equal* | Reference |  |  |  |  |
|  | *Decreased* | 0.126 | .860 | 1.134 | 0.281 | 4.581 |
| **Dream Vividness** | |  |  |  |  |  |
|  | *Increased* | 0.301 | .608 | 1.351 | 0.428 | 4.262 |
|  | *Equal* | Reference |  |  |  |  |
|  | *Decreased* | 0.080 | .905 | 1.084 | 0.290 | 4.05 |
| **Mood** | | 0.035 | .853 | 1.036 | 0.714 | 1.502 |
| **Stress** | | 0.578 | .169 | 1.782 | 0.782 | 4.061 |
| **General Fear** | | 1.057 | **.013** | 2.878 | 1.255 | 6.602 |
| **Fear of contagion** | | 0.175 | .630 | 1.191 | 0.584 | 2.43 |
| **Δ PSQI** | | -0.025 | .669 | 0.975 | 0.869 | 1.094 |
| **Δ Sleep Midpoint** | | -0.266 | .132 | 0.767 | 0.543 | 1.083 |
| **Δ Emotional Dream Valence** | | 0.353 | .112 | 1.424 | 0.921 | 2.201 |
| **Overal model test:** χ2_15_=26.90, p=.030, R^2^_N_=.174.  *Notes:* n=214; PSQI: Pittsburgh Sleep Quality Index; R^2^_N_: Nagelkerke’s R^2^ | | | | | | |
